# CD4^+^ T effector memory cell responses in *Chlamydia pneumoniae*‐stimulated peripheral blood mononuclear cells in nonasthmatic subjects

**DOI:** 10.1002/iid3.726

**Published:** 2022-10-27

**Authors:** Tamar A. Smith‐Norowitz, Sarah Shidid, Yitzchok M. Norowitz, Stephan Kohlhoff

**Affiliations:** ^1^ Department of Pediatrics, Division of Infectious Diseases State University of New York Downstate Health Sciences University Brooklyn New York USA

**Keywords:** CD4, *Chlamydia pneumonia*, T effector memory cells

## Abstract

*Chlamydia pneumoniae* (*C. pneumoniae*) is a gram‐negative intracellular bacterium that causes respiratory infection in humans, including subjects with or without asthma. *C. pneumoniae* activates cells (e.g., monocytes/macrophages) in vitro, and produces cytokines that may contribute to inflammatory responses observed in asthma. Immunological differences exist between subjects with or without asthma, with regard to host responses to *C. pneumoniae*. The heterogeneity and subsequent diverse pathophysiology of asthma can be better understood by analyzing the repertoire of T‐cell subpopulations; the most common distinction between different asthma endotypes includes cytokines produced by CD4^+^ cells (T helper (Th)2 high vs. Th2 low).

1

*Chlamydia pneumoniae* (*C. pneumoniae*) is a gram‐negative intracellular bacterium that causes respiratory infection in humans,^1^, ^2^ including subjects with or without asthma.^2^, ^3^, ^4^, ^5^ *C. pneumoniae* activates cells (e.g., monocytes/macrophages) in vitro, and produces cytokines that may contribute to inflammatory responses observed in asthma.^2^, ^6^ Immunological differences exist between subjects with or without asthma, with regard to host responses to *C*. *pneumoniae*.^7^ The heterogeneity and subsequent diverse pathophysiology of asthma can be better understood by analyzing the repertoire of T‐cell subpopulations^8^; the most common distinction between different asthma endotypes includes cytokines produced by CD4^+^ cells (T helper (Th)2 high vs. Th2 low).^8^

Prior literature demonstrated that *C. pneumoniae*‐stimulated peripheral blood mononuclear cells (PBMC) induce predominantly Th2 responses and Immunoglobulin (Ig) E,^7^ which may indicate chronic inflammation due to persistent infection. Others reported that *C. pneumoniae*‐induced interferon (IFN)‐gamma production (Th1 response) in vitro was more prevalent in children with asthma than nonasthma,^9^ and is consistent with presence of circulating T effector memory cells (TEMS).^9^ Circulating memory cells responsive to chlamydial antigen are found in humans.^10^ Following *C. pneumoniae* infection, TEMS may play a role in immune‐protection and pathology; these cells may indicate either past or persistent infection.^11^ Smith‐Norowitz et al.^11^ demonstrated in healthy individuals, that *C. pneumoniae*‐induced PBMC IFN‐gamma+ responses increased numbers of CD4^+^ IL‐2^+^ and CD4^+^ IL‐4^+^ TEMS, while CD8^+^ IL‐2^+^ and CD8^+^ IL‐4^+^ TEMS decreased. However, little is known about the role of *C. pneumoniae*‐specific TEMS in humans^10^; pathogen‐specific TEMS may have a key role in immune control of persistent virus infection.^10^ The aim of this study sought to better understand the prevalence and define the characteristics of CD4^+^ TEMS in a healthy population that can serve as a comparison cohort for patients with asthma or other respiratory conditions that may be adversely affected by *C. pneumoniae* infections.

Subjects were recruited from an outpatient primary care practice (Brooklyn, NY). PBMC (1 × 10^6^/ml) from adult nonasthmatic subjects (*N* = 5; 3 females, 2 males, ages 24–65) were infected for 1h^+/−^ *C. pneumoniae* TW‐183 (ATCC Catalog No. VR2282; ATCC) at a multiplicity of infection (MOI) = 0.1. Two different stimulation conditions were used (1:10 and 1:100). Cells were cultured (48h), as previously described.^11^ All patients were White, and nonasthmatic. Single‐and dual‐color immunophenotyping of lymphocytes was performed, with modifications for intracellular staining for cytokines.^11^ Distributions of lymphocytes and TEMS (CD4^+^, CD8^+^, CD45RA^+^, CD45RO^+^, CCR7^+^, CD154^+^) and intracellular cytokine (IFN‐gamma) were determined (flow microfluorimetry) (Coulter Epics XL, MCL Flow Cytometer; Coulter), as previously described.^11^ TEMRA are effector memory T cells that re‐express CD45RA, and TCM are central memory T cells. TEM totals are TEMS and TEMRA cells. Statistical analyses were performed in SAS v9.4.3. This study was conducted in accordance with the guidelines and Declaration of Helsinki and approved by the SUNY Downstate Health Sciences University institutional review board (IRB number 370511‐12). Patient consent was obtained.

CD4^+^ T effector memory cell responses in *C. pneumoniae*‐stimulated PBMC are shown in Table 1. No statistical significances were observed between cell subsets listed (CD4^+^ TCM, CD4^+^ TEMRA, CD4^+^ TEM Total, TEM IFN‐gamma, TEMRA IFN‐gamma, TEM Total IFN‐gamma) (*p* = 0.202, 1.00, 0.779, 0.368, 0.717, 0.223, respectively) for all stimulation conditions (unstimulated, 1:10, or 1:100) (Friedman *χ* ^2^ test).

**Table 1:** CD4^+^ T effector memory cell responses in *Chlamydia pneumoniae*‐stimulated PBMC in nonasthmatic subjects

Lymphocyte subset	Stimulation condition			*p* Value^a^
(% total)	Unstimulated	1:10	1:100	
CD4^+^ TCM	0.10 ± 0.14	0.15 ± 0.13	0.08 ± 0.10	0.202
CD4^+^ TEMRA	8.2 ± 8.79	5.9 ± 2.59	6.0 ± 6.61	1.000
CD4^+^ TEM total	21.2 ± 19.09	19.6 ± 7.50	19.9 ± 8.50	0.779
TEM IFN‐gamma	0.03 ± 0.05	0 ± 0	0.03 ± 0.05	0.368
TEMRA IFN‐gamma	0.03 ± 0.05	0.05 ± 0.06	0.05 ± 0.06	0.717
TEM total IFN‐gamma	0.25 ± 0.37	0.05 ± 0.06	0.075 ± 0.05	0.223

*Note*: *C. pneumoniae*‐infected PBMC, 1:10 and 1:100. MOI: 0.1.

Abbreviations: IFN, interferon; MOI, multiplicity of infection; PBMC, peripheral blood mononuclear cells; TCM, central memory T cells; TEM, effector memory T cells; TEMRA, effector memory T cells re‐express CD45RA; TEM total, TEMs and TEMRAs.

Data represented as mean (% total) +/− standard deviation; five representative nonasthmatic subjects.

^a^ Friedman *χ* ^2^ test.

T cells (cell‐mediated immunity) and their role in viral infection are well recognized^12^; virus‐specific CD4^+^ and CD8^+^ T cells kill virus‐infected cells and also produce cytokines.^13^ Virus‐specific CD4^+^ T‐cell responses are characterized by Th1 responses (i.e., IFN‐gamma production).^13^ Rothfuchs et al.^14^ demonstrated in a murine model that macrophages, CD4^+^ and CD8^+^ T cells play a protective role against *C. pneumoniae* and other chlamydial species through their ability to secrete interferon gamma. These cells enhanced immune protection against *C. pneumoniae*.^14^ Virus‐specific TEMS have been described in other persistent infections (i.e., Epstein Barr virus, Adenovirus, HIV).^11^

This study examines different lymphocyte subpopulations in a *C. pneumoniae*‐infection PBMC model. These findings show that numbers of TEMS, TEMRAs or TCMs did not change between stimulation conditions in this model system. The results may indicate low prevalence of *C*. *pneumoniae‐*specific circulating memory cells in healthy subjects. Bunk et al demonstrated that memory CD4^+^ T cells responding to *C. pneumoniae* stimulation can be detected in circulation from healthy subjects.^10^ The presence or absence of dual IFN‐gamma and IL‐2 producing CD4^+^ T‐cell responses was associated with different patterns of IgG reactivity toward *C. pneumoniae* antigens during persistent infection.^10^ However, the current findings are not consistent with a significant prevalence of *C. pneumoniae* responsive CD4^+^ memory T cells at the time of our study and in the population studied. This demonstrates the importance of identifying representative control populations when studying infection and disease associations. Study limitations include small sample size of the cohort. It should be mentioned that the absence of significant differences could be linked to the MOI used; similarly, the short infection time could also affect the results. Future examination of these cell populations in larger samples of adults or children, and different disease states (i.e., asthma), is warranted.

## AUTHOR CONTRIBUTIONS

**Tamar Smith‐Norowitz**: Conceptualization; data curation; formal analysis; investigation; methodology; project administration; supervision; validation; visualization; writing – original draft preparation; writing – review and editing. **Stephan Kohlhoff**: Conceptualization; data curation; formal analysis; investigation; methodology; supervision; visualization; writing – review and editing. **Yitzchok Norowitz**: Data curation; formal analysis; investigation; methodology. **Sarah Shidid**: Data curation; formal analysis; investigation; methodology.

## CONFLICT OF INTEREST

The authors declare no conflict of interest.

## ETHICS STATEMENT

This study was approved by the SUNY Downstate Health Sciences University review board.

## Data Availability

Data available on request from the authors.
